# MiR-149-5p: An Important miRNA Regulated by Competing Endogenous RNAs in Diverse Human Cancers

**DOI:** 10.3389/fonc.2021.743077

**Published:** 2021-10-15

**Authors:** Fu-jia Ren, Yao Yao, Xiao-yu Cai, Yu-ting Cai, Qian Su, Guo-ying Fang

**Affiliations:** ^1^ Department of Pharmacy, Hangzhou Women’s Hospital (Hangzhou Maternity and Child Health Care Hospital), Hangzhou, China; ^2^ Department of Pharmacy, Women’s Hospital School of Medicine, Zhejiang University, Hangzhou, China; ^3^ Department of Clinical Pharmacology, Key Laboratory of Clinical Cancer Pharmacology and Toxicology Research of Zhejiang Province, Affiliated Hangzhou First People’s Hospital, Zhejiang University School of Medicine, Hangzhou, China

**Keywords:** MiR-149-5p, human cancers, reproductive system, digestive system, respiratory system

## Abstract

MicroRNAs (miRNAs) consist of a large family of small, non-coding RNAs with the ability to result in gene silencing post-transcriptionally. With recent advances in research technology over the past several years, the physiological and pathological potentials of miRNAs have been gradually uncovered. MiR-149-5p, a conserved miRNA, was found to regulate physiological processes, such as inflammatory response, adipogenesis and cell proliferation. Notably, increasing studies indicate miR-149-5p may act as an important regulator in solid tumors, especially cancers in reproductive system and digestive system. It has been acknowledged that miR-149-5p can function as an oncogene or tumor suppressor in different cancers, which is achieved by controlling a variety of genes expression and adjusting downstream signaling pathway. Moreover, the levels of miR-149-5p are influenced by several newly discovered long non-coding RNAs (lncRNAs) and circular RNAs (circRNAs). However, there is blank about systematic function and mechanism of miR-149-5p in human cancers. In this review, we firstly summarize the present comprehension of miR-149-5p at the molecular level, its vital role in tumor initiation and progression, as well as its potential roles in monitoring diverse reproductive and digestive malignancies.

## 1 Introduction

It is widely acknowledged that microRNAs (miRNAs), a type of short (~22 nucleotides), single-stranded non-coding RNA, are reported to regulate cellular proliferation, differentiation, apoptosis, oxidative stress, and autophagy through binding to the 3′-untranslated region (UTR) of target mRNA, thereby causing translational repression or mRNA degradation in animals or plants (1–4). The process of mature miRNA formation requires the cooperation and coordination of various enzymes and proteins. In humans, four vital enzymes, including Drosha, exportin 5, Dicer and argonaute 2 (AGO2), participate in miRNA processing (5). Unfortunately, increasing evidence indicate that cancers may occur when this finely coordinated processing is disrupted, or when one or more of the enzymes are mutated, which consequently leads to oncogene awakening or tumor suppressor gene silencing (6). Historically, one study from Carlo Croce’s laboratory identified that miRNAs play an important role in cancer initiation and progression. This pathbreaking study reported that miR-15/16 acts as tumor suppressors in chronic lymphocytic leukemia (CLL), promoting numerous researchers to unveil the non-negligible role of miRNA in cell proliferation, migration, metastasis, energy metabolism of various cancers (7). The current studies have provided evidences that the activity of miRNAs can be influenced by the existence of competing endogenous RNAs (ceRNAs), vying for the miRNAs with shared miRNAs responses elements(MREs) (8). Moreover, with the rapid development of high-throughput sequencing technology, molecular biology and life science, other ceRNAs apart from protein-coding ceRNAs, such as lncRNAs and circRNAs, sharing common MRE, are discovered as important upstream modulators by serving as miRNA sponges, thereby repressing normal miRNA targeting activity on mRNA (9, 10). Consequently, advances in keeping miRNAs as a balanced level *in vivo* through adjusting the levels of ceRNAs, utilizing miRNA inhibitor or mimics delivery methods, such as nanoparticle delivery systems and exosome carrier delivery systems, will make miRNA-based therapeutics feasible (11, 12). Overall, miRNAs have promising therapeutic and predictive potentials in cancers treatment.

Recently, mounting evidences have indicated the pleiotropic functions of miR-149-5p in different human cancers. MiR-149-5p serves as tumor suppressor in several cancers by targeting specific mRNA expression, such as gastric cancer (GC) (13), breast cancer (14), hepatocellular carcinoma (HCC) (15), and colorectal cancer (CRC) (16), whereas in lung adenocarcinoma (LUAD) (17), acute lymphoblastic leukemia (ALL) (18), and acute myeloid leukemia (AML) (19), it acts as oncomiR, promoting tumorigenesis and aggression (20). Intriguingly, miR-149-5p was reported to be regulated by lncRNAs and circRNAs in cancer development, such as LINC00460 in CRC, CircNRIP1 in GC, and hsa_circ_0075341 in cervical cancer (13, 16, 21–23). Yet, the function and mechanism of miR-149-5p in cancer initiation and progression have not been fully understood. In this review, we systematically summarize the expression, function, target genes, upstream regulators and application potentials in different cancers, with special emphasis on reproductive system and digestive system cancers.

## 2 Physiological Roles of miR-149-5p

### 2.1 Role of miR-149-5p in Adipogenesis

A recent study by Khan’s team analyzed the expression of miR-149-5p during adipogenesis and found that miR-149-5p was highly expressed in bovine adipocytes on the 9th day of proliferation and differentiation. Functional studies have shown that miR-149-5p inhibits the proliferation and differentiation of bovine adipocytes by targeting CRTC1 and CRTC2, two well-known regulators of adipogenesis (24). The team further found that miR-149-5p plays a role in adipogenesis through cross-regulation of differential expressed genes, such as CCND2, KLF6, ACSL1, Cdk2, SCD, SIK2, and ZEB1, and its respective KEGG pathways in bovine adipocytes. To sum up, their results suggest that miR-149-5p can regulate the lipid metabolism of bovine adipocytes (25).

The adipocytes perform various roles, particularly energy storage in the form of triglycerides. Intriguingly, increasing studies have indicated that some adipocytes, as an important element of the stromal microenvironment in various cancers, exhibit tumor-promoting effects on different tumor cells by influencing excretion of adipokines and proinflammatory cytokines, thus this group of adipocytes is characterized as cancer-associated adipocytes (CAAs). Recently, increasing miRNAs have been found to play a crucial role in communication between CAAs and tumor cells (26). For example, Wu et al. revealed that breast cancer cells co-cultured with mature adipocytes display an aggressive phenotype through enhancing epithelial-mesenchymal transition (EMT). Mechanistically, exosome-derived miR-155 originated from breast cancer cells promoted beige/brown differentiation and remodel metabolism in resident adipocytes (27). Notably, miR-149-3p was reported to participate in a subcutaneous-to-visceral fat switch during 24 h fasting. Mechanistically, in cultured inguinal preadipocytes, overexpression of miR-149-3p promoted a visceral-like switch during cell differentiation (28). However, it has not been reported whether miR-149-5p is involved in differentiation, proliferation or cytokine release of human adipocytes. Given the role of miR-149-5p in bovine adipogenesis, it is necessary to study whether miR-149-5p is involved in activity of adipocytes, especially CAAs in human cancers.

### 2.2 Role of miR-149-5p in Vascular Smooth Muscle Cells

Vascular smooth muscle cells (VSMCs) are class of cells maintaining normal vascular structure, but they will manage their ability to migrate to the intima and proliferate to supplement neointimal lesions when pathological damages occurs (29). Zhang et al. found that the levels of miR-149-5p are down-regulated in PDGF-BB-induced VSMCs in a time- and dose-dependent manner. Overexpression of miR-149-5p can inhibit the proliferation, invasion and migration of VSMCs, while miR-149-5p knockdown has the opposite effect. In addition, histone deacetylase 4 (HDAC4) has been found to be a potential target for miR-149-5p, which can rescue the repressed effects on VSMCs mediated by miR-149-5p (30). Consistent with the above results, Peng et al. found that circDHCR24, a sponge for miR-149-5p, increased the expression of MMP9, which in turn promoted the proliferation, migration and phenotypic transformation of human VSMCs (31). Besides, Wang et al. found that circ CHFR regulates the expression of neuropilin 2 (NRP2) *via* sponging miR-149-5p, subsequently promoting the proliferation, migration and invasion of human VSMCs (32). In short, miR-149-5p performs biological functions through various pathways and may provide effective therapeutic potentials for VSMC growth-related diseases.

### 2.3 Other Functions

Wang et al. found that the levels of miR-149-5p in the skin tissue of superior-quality brush hair goats are higher than that of ordinary brushed goats. Functional studies indicated miR-149-5p plays an important role in the formation of high-quality hair traits. Through the post-transcriptional mechanism, it can inhibit the expression of CKLF-like MARVEL transmembrane domain-containing 3 (CMTM3), promote the proliferation of goat hair follicle stem cells and inhibit the apoptosis of hair follicle stem cells (33).

## 3 MiR-149-5p in Human Cancers

### 3.1 Cancers of the Reproductive System

#### 3.1.1 Breast Cancer

As we all know, breast cancer is the most common malignant tumor in female reproductive system. According to the global cancer statistics in 2018, there are about 2.1 million newly diagnosed cases of female breast cancer, accounting for 1/4 of female cancer, which is a serious threat to the health of women around the world (34).

Fortunately, many of the recent and upcoming anticancer drugs are attempted to treat breast cancer. It has been reported that paclitaxel (PTX) is a first-line drug for the treatment of breast cancer. However, due to the resistance of breast cancer to PTX, the application of PTX is limited (35). Recently, a study by Xiang et al. revealed that ursolic acid (UA), a pentacyclic triterpenoid existing widely in plant, can reverse the resistance of MDA-MB-231, one breast cancer cell line, to PTX. The expression of miR-149-5p in 231/PTX cells treated with UA was remarkably higher compared to untreated 231/PTX cells. Intriguingly, the reverse effect of UA disappeared after miR-149-5p inhibitors treatment, indicating that UA has a reversal effect by up-regulating the expression of miR-149-5p. Bioinformatics analysis showed that there was a binding site of miR-149-5p in the 3’-UTR of MyD88. Furthermore, the reversal effect of UA in 231/PTX cell disappeared after overexpression of MyD88, suggesting that miR-149-5p negatively regulate the expression of MyD88 by binding to its 3’-UTR, thereby reversing drug resistance of breast cancer (36). The protective effect of miR-149-5p was also proved in trastuzumab resistance (Tr-R). At present, trastuzumab is the first choice for treating HER2-overexpressing breast cancer by restraining HER2 expression, but its drug resistance is gradually increasing (37). Tian and coworkers found that propofol, a common intravenous anesthetic, has anti-cancer effects in breast cancer *via* epigenetically upregulating miR-149-5p expression in HER2-overexpressing cell with Tr-R (38). In short, miR-149-5p might be a target for reversing drug resistance in breast cancer, and finding an upstream regulator of miR-149-5p is also important for comprehending the intrinsic mechanism. Qi and collaborators found that circ_0072995 promotes breast cancer cell progression by acting as a sponge for miR-149-5p, thereby upregulating serine hydroxymethyltransferase 2 (SHMT2), an oncogene in breast cancer (39). Later, Temiz et al. found that in breast cancer, overexpression of miR-149-5p reduces the expression of Chaperonin Containing TCP1 Subunit 3 (CCT3), which leads to the destruction of intracellular reactive oxygen species (ROS) homeostasis and the distribution of free amino acids in energy metabolism, and promotes tumor cell apoptosis (14).

**Figure 1:**
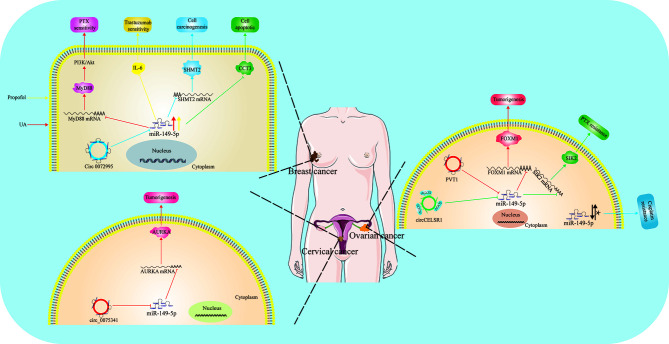
Function and regulatory mechanism of miR-149-5p in reproductive system. MiR-149-5p plays an anticancer role in breast cancer and cervical cancer by targeting important genes. However, its role in ovarian cancer remains controversial (*). The expression of miR-149-5p is regulated by non-coding RNA, including circ_0072995, circ_0075341, circCELSR1 and circPVT1.

Collectively, miR-149-5p may act as a tumor suppressor and restoring the expression of miR-149-5p in breast cancer may be an effective strategy for breast cancer treatment (**Figure 1**).

#### 3.1.2 Ovarian Cancer

Ovarian cancer is the third most common gynecological malignant tumor in the world, but it has the highest mortality among these cancers. Globally, there are 239000 new cases and 152000 deaths each year (40). The treatment of ovarian cancer, especially advanced and recurrent ovarian cancer, has always been the biggest challenge in clinical work.

Xu et al. found that the expression of miR-149-5p in chemotherapy-resistant ovarian cancer tissues is abnormally higher than that in chemotherapy-sensitive ovarian cancer tissues and non-cancer tissues. Functionally, silencing of miR-149-5p enhanced the sensitivity of ovarian cancer cells to cisplatin *in vivo* and *in vitro*. On the contrary, overexpression of miR-149-5p increased the chemotherapy resistance of ovarian cancer cells. Mechanistical studies showed that miR-149-5p directly inhibits MST1 and SAV1, two key proteins in Hippo pathway, thereby leading to inactivation of Hippo signals and promoting chemotherapy resistance of ovarian cancer cells to cisplatin (41).

Paradoxically, Sun et al. found that the expression of miR-149-5p is down-regulated in chemotherapy-resistant ovarian cancer tissues and cells, and the overexpression of miR-149-5p inhibited the growth of ovarian cancer cells and promoted apoptosis and cisplatin sensitivity (42). Consistently, Wei et al. found that compared with PTX-sensitive ovarian cancer tissues and cells, the expression of miR-149-5p was down-regulated in PTX-resistant ovarian cancer tissues and cells. And overexpression of miR-149-5p could enhance the sensitivity of PTX, inhibit cell viability and clone formation, block cell cycle, and induce apoptosis of PTX-resistant cells. In addition, inhibition of miR-149-5p could effectively reverse the effects of circ_CELSR1 deficiency on PTX resistance, cell survival, colony formation, cell cycle and apoptosis of PTX-resistant cells, indicating that circ_CELSR1 increased PTX resistance of PTX-resistant cells through acting sponges for miR-149-5p. The up-regulation of salt inducible kinase 2 (SIK2) reversed the inhibitory effect of miR-149-5p on PTX resistance and cell progression in PTX-resistant ovarian cancer cells, indicating that miR-149-5p enhanced PTX sensitivity by targeting SIK2. In summary, they found that circ_CELSR1 can act as a sponge for miR-149-5p, thus regulating the expression of SIK2 (43). Moreover, Li et al. found that the expression of PVT1 increased in ovarian cancer, and circular PVT1 regulated miR-149-5p in the form of ceRNA, and then up-regulated Forkhead Box M1 (FOXM1), subsequently promoting the occurrence of ovarian cancer (44).

Conclusively, the emerging role of miR-149-5p is gradually investigated (**Figure 1**), but due to the contrary opinions, the function of miR-149-5p in ovarian cancer needs to be further studied.

#### 3.1.3 Cervical Cancer

Cervical cancer is a growing global burden for both developing and developed countries. It is estimated that there were 570000 cases and 311000 deaths worldwide in 2018, making it the fourth most commonly diagnosed cancer among women and the fourth leading cause of cancer death among women (34).

Based on microRNA sequencing and bioinformatics analysis, miR-149-5p was found as a potential regulatory factor in HPV-positive cervical squamous cell carcinoma (45). Recently, Shao et al. found that the expression of hsa_circ_0075341 is aberrantly up-regulated in patients with cervical cancer, which was related to tumor size, FIGO stage progression and lymph node metastasis. Inhibition of hsa_circ_0075341 *in vitro* weakened the proliferation and invasion of cervical cancer cells. Circinteractome analysis confirmed that hsa_circ_0075341 may have a target site for miR-149-5p, which was further verified by luciferase report experiment and qRT-PCR. Their study found that the expression of miR-149-5p is down-regulated in cervical cancer, which is related to poor prognosis. Rescue assay showed that inhibition of miR-149-5p can block the effect of circ_0075341 siRNA on the proliferation of cervical cancer cells. Therefore, hsa_circ_0075341 may act as a sponge for miR-149-5p in cervical cancer. In addition, they found that increased expression of Aurora kinase A (AURKA) was associated with poor prognosis in patients with cervical cancer. Functional analysis showed that inhibition of AURKA could reduce the proliferation and invasion of cervical cancer cells *in vitro*. In terms of mechanism, they further confirmed that AURKA is the target for miR-149-5p. Therefore, miR-149-5p may act as key modulator in the oncogenic role of hsa_circ_0075341 in cervical cancer, by binding 3’-UTR region of AURKA (23) (**Figure 1**). Besides, Liu et al. found that hsa_circ_0061140 may play a role in promoting tumorigenesis in endometrial carcinoma by inducing the expression of STAT3 as a molecular sponge for miR-149-5p (46). Collectively, miR-149-5p may be a tumor suppressor in cervical cancer progression and further studies are needed to confirm its therapeutic effect.

#### 3.1.4 Prostate Cancer

Prostate cancer (PCa) is the most common non-skin cancer in men in the world. It is estimated that there are nearly 1.3 million new cases of PCa and 359000 related deaths worldwide in 2018, making it the second most common cause of cancer and the fifth leading cause of cancer death among men (34).

In 2019, Fu et al. found the anticancer effect of Fuzheng Yiliu decoction (FZYL), a Chinese medicinal formulae, combined with docetaxel (Doc) was enhanced in PCa, compared with one of them in a castration-resistant prostate cancer (CRPC) mouse model. By analyzing the differential miRNA in tumor tissues treated with Doc + FZYL, there were 10 specific miRNAs, in which miR-149-5p sharply decreased. The enrichment analysis of speculated target genes by KEGG and GO showed that Doc + FZYL-specific miRNAs may be involved in PI3K-Akt signal pathway to enhance the therapeutic effect (47).

Subsequently, Ma et al. found that the expression of miR-149-5p is down-regulated in PCa, and overexpression of miR-149-5p weakened the malignant degree of PCa cells by regulating Regulator of G Protein Signaling 17 (RGS17). Mechanistically, silencing of miR-149-5p upregulated the expression of RGS17 in PCa tissues and cells, while overexpression of miR-149-5p showed an opposite effect (48). Besides, Temiz et al. found that in PCa cell lines, overexpression of miR-149-5p can downregulate the expression of CCT3, which leads to the destruction of intracellular ROS homeostasis and the distribution of free amino acids in energy metabolism, and promotes tumor cell apoptosis (14).

Overall, miR-149-5p may be a vital tumor suppressor in reproductive system cancers (**Figure 1**), but its function in ovarian cancer is controversial. To explore the diagnostic role in reproductive system cancers, essential studies are needed to perform.

### 3.2 Cancers of the Digestive System

#### 3.2.1 Gastric Cancer

Gastric cancer is a dangerous disease in the world, which threatens human lives. It is estimated that there are more than 1 million new cases of GC every year, making it the fifth largest disease diagnosed as a malignant tumor in the world. Because GC is often diagnosed at an advanced stage and has a high mortality rate, it ranked the third most common cause of cancer-related deaths, with 784000 deaths worldwide in 2018 (49).

The discovery of the role of miR-149-5p in GC is attributed to the studies on circRNA and lncRNA. Recently, the next-generation sequencing method was used to detect the differentially expressed circRNA in GC tissues. It was found that CircNRIP1 was up-regulated exponentially in GC tissues compared with adjacent normal gastric tissues, and the levels of CircNRIP1 were significantly correlated with the size of GC, lymphatic invasion, disease-free survival and overall survival. Functionally, CircNRIP1 knockdown successfully blocked the proliferation, migration, invasion and AKT1 expression of GC cells. Interestingly, miR-149-5p inhibited the oncogenic role of CircNRIP1 in GC cells, and the overexpression of miR-149-5p blocked the malignant behavior of CircNRIP1. Experiments *in vitro* suggested that CircNRIP1, as a sponge for miR-149-5p, promotes the progression of GC through AKT/mTOR-mediated metabolism and EMT pathway. In addition, CircNRIP1 can be transmitted between GC cells through exosome communication, and CircNRIP1 in exosomes can also promote tumor metastasis *in vivo*. Further studies on the mechanism show that RNA binding protein QKI can promote the cyclic transcription of NRIP1 gene. Finally, the tumor promoting effect of CircNRIP1 was verified in patient-derived xenograft (PDX) model (13).

Moreover, another study demonstrated that lncRNA BLACAT1 promotes the proliferation, migration and invasion of GC cells by regulating the miR-149-5p/KIF2A axis (50). CircNHSL1 can affect the expression of YWHAZ through sponging miR-149-5p, thereby regulating the progression of GC (51). Circ_0044516 could promote the expression of HuR through sponging miR-149-5p, thereby regulating the progression of GC (52). Importantly, Chen et al. reported low serum levels of miR-149-5p, combined with miR-1-3p, miR-125b-5p and miR-196a-5p, may be used as a noninvasive biomarker for gastric adenocarcinoma diagnosis (53). Given the crucial role of miRNA in cellular communication through exosome, it is necessary to explore whether miR-149-5p exists in exosome in GC.

**Figure 2:**
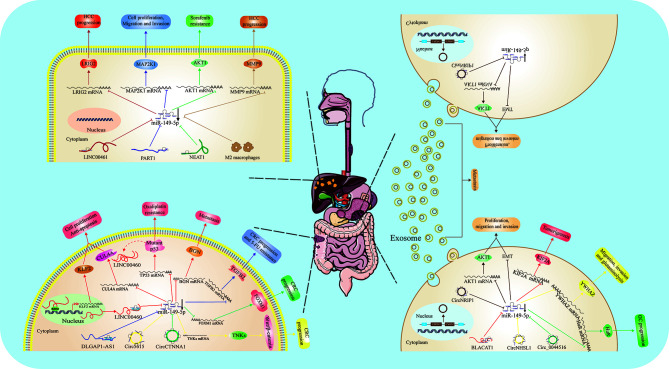
Function and regulatory mechanism of miR-149-5p in digestive system. MiR-149-5p inhibits cell proliferation, migration, invasion and drug resistance in HCC, GC and CRC through binding 3’-UTR of target mRNA. In disease state, miR-149-5p is usually imprisoned by some noncoding RNAs, including LINC00461, PART1, NEAT1 in HCC; LINC00460, DLGAP1-AS1, circ5616 and circCTNNA1 in CRC; BLACAT1, circNRIP1, circNHSL1 and circ_0044516 in GC. In GC, circNRIP1 exists in exosome, and mediates cell to cell communication.

Collectively, as a tumor suppressor in GC, miR-149-5p expression was imprisoned by a variety of lncRNAs and circRNAs (**Figure 2**). Therefore, targeting some upstream non-coding RNAs is a good choice to rescue the normal levels of miR-149-5p.

#### 3.2.2 Hepatocellular Carcinoma

Liver cancer is predicted to be the fourth leading cancer-related deaths and the sixth most commonly diagnosed cancer worldwide in 2018, posing a serious threat to human quality of life (34). Based on annual data, the World Health Organization estimates that more than 1 million people will die of liver cancer in 2030 (54). Hepatocellular carcinoma is the most common tumor in all primary liver cancer, accounting for 80% of the total number of cases (55).

In HCC, miR-149-5p was also regarded as tumor suppressor, and was regulated by several important lncRNAs. For example, Ji et al. found that LINC00461 was a ceRNA by directly sponging miR-149-5p in liver cancer cells, and then regulated LRIG2, to play a carcinogenic role (56). Zhou et al. found that lncRNA PART1 promotes the proliferation, migration and invasion of HCC cells by regulating the miR-149-5p/MAP2K1 axis (57). Moreover, Chen et al. confirmed that miR-149-5p was associated with lung metastasis in patients with liver cancer (58).

Niu et al. found that the expression of NEAT1 in HCC tissues was significantly higher than that in adjacent tissues. Functional analysis showed that NEAT1 could directly bind to miR-149-5p, leading to suppression of miR-149-5p/AKT1 axis, thereby promoting the drug resistance of HCC cells to sorafenib (59).

It is widely acknowledged that tumor microenvironment (TME) plays a crucial role in tumor growth, development and metastasis, among which inflammation is one of the most important factors, and macrophages are class of the most common immune-related cells in it. Macrophages can be classified into two types, classical macrophages (M1) and alternative (M2) macrophages, in microenvironment. In general, M1 macrophages play a pro-inflammatory role by expressing nitric oxide synthase (INOS), while M2 macrophages express anti-inflammatory cytokines, such as IL-10, to promote tumor progression and metastasis (60). Liu et al. found that M2 macrophages may increase the expression of MMP9 by reducing the level of miR-149-5p in HCC cells and promote the progression of HCC (15), further confirmed the tumor suppressive role of miR-149-5p in HCC. Interestingly, increasing evidences revealed that hypoxia can regulate the status of tumor immune microenvironment, such as promoting the release of inflammation factors, enhancing the recruitment of innate immune cells (61). Thus, it is meaningful for researchers to study whether hypoxia alters the level of miR-149-5p and whether miR-149-5p is involved in the changes of immune microenvironment induced by hypoxia in HCC.

In order to accurately predict the prognosis of HCC patients, many teams have constructed a model containing miRNAs (62–64). Inconsistent with above mentioned studies (**Figure 2**), they reported that miR-149-5p was one of the elements, and its high expression was associated with poor prognosis. Therefore, further studies should pay more attention on clinical samples, and analyze the correlation between miR-149-5p with HCC.

#### 3.2.3 Colorectal Cancer

A few decades ago, CRC was rarely diagnosed. According to 2018 GLOBOCAN, CRC ranks third in terms of incidence, but second in terms of mortality. Over 1.8 million new CRC cases and 881,000 deaths are estimated to occur in 2018, accounting for about 1 in 10 cancer cases and deaths (34). The incidence and mortality of CRC vary geographically, with the highest rates in developed countries, but with the continuous progress of developing countries, the global incidence of CRC is expected to increase to 2.5 million new cases by 2035 (65).

By analyzing TCGA RNA sequencing data and other publicly available microarray data, Lian et al. found a new lncRNA, LINC00460, whose expression in CRC tissues is significantly higher than that in adjacent normal tissues. Importantly, the high levels of LINC00460 in CRC were associated with larger tumor, advanced tumor stage, lymph node metastasis and shorter overall survival. Functional studies indicated LINC00460 can promote proliferation and inhibit apoptosis of CRC cells *in vitro* and *in vivo.* Mechanistical studies demonstrated that LINC00460 can be served as a molecular sponge for miR-149-5p, antagonizing its capacity to inhibit cullin 4A (CUL4A) protein translation, which suggests a tumor suppressive role of miR-149-5p, in contrast to the oncogenic function of LINC00460 in CRC (16). Consistently, Ruan et al. confirmed that LINC00460 can be used as the ceRNA for miR-149-5p to up-regulate BGN, thus promotes the metastasis of CRC cells (22).

It has been reported that oxaliplatin resistance was a major challenge in clinical treatment of advanced CRC (66). Meng et al. reported that LINC00460-miR-149-5p/miR-150-5p-mutant p53 feedback loop was associated with oxaliplatin resistance in CRC. LINC00460 was exhibited a high level in oxaliplatin-resistant CRC (CRC/OxR) cells than that in oxaliplatin-sensitive CRC cells, and this expression pattern depends on mutant p53 (SW480/OxR) rather than wild-type p53 (HCT116/OxR). Further studies suggested that LINC00460 promotes oxaliplatin resistance by inhibiting miR-149-5p/miR-150-5p, and improving the expression of miRNA-targeted p53 (21). Moreover, Qu et al. found that lncRNA DLGAP1-AS1 knockdown inhibits the occurrence of CRC by regulating miR-149-5p/TGFB2/Smad2 signal pathway *in vivo* and *in vitro*, and improves the sensitivity of 5-FU (67). These studies highlighted an important role of miR-149-5p in CRC, and the intimate regulatory relation between miRNA-149-5p and lncRNAs.

It is worth mentioning that the levels of miR-149-5p are also regulated by two newly discovered circRNAs, Circ5615 and CircCTNNA1. The study by Ma et al. first explored the expression profile of circRNA in 5 pairs of CRC tissues by microarray, and found that one CircRNA, hsa_CIRC_0005615 (Circ5615) was remarkably up-regulated in CRC tissues. The upregulation is closely related to the high T stage and poor prognosis of patients with CRC. Studies *in vitro* and *in vivo* have shown that in CRC cells, Circ5615 gene knockdown inhibited cell proliferation and cell cycle acceleration, while overexpression promoted malignant phenotype. Mechanism studies have shown that Circ5615, as a sponge for miR-149-5p, inhibits miRNA-mediated inhibition of target gene TNKS. The increase of TNKs level can stabilize β-catenin by stimulating the degradation of AXIN2. Promote the proliferation of CRC cells through Wnt/β-catenin pathway (68).

Similarly, Chen et al. used CircRNA and mRNA microarray techniques to analyze the colon cancer tissues and paracancerous normal tissues of 3 patients with colon cancer. The most related mRNA (FOXM1) and CircRNA (CircCTNNA1, a new CircRNA) were significantly up-regulated in colon cancer, and their levels were related to the stage of lymph node metastasis, poor prognosis and poor survival in patients with colon cancer. CircCTNNA1 can promote the proliferation, migration and invasion of colon cancer cells *in vitro* and *in vivo*. Functional analysis showed that CircCTNNA1 could be used as a ceRNA of miR-149-5p to counteract the inhibitory effect of miR-149-5p on downstream target gene FOXM1 (69).

Collectively, miR-149-5p exerts a tumor suppressive role in CRC, and is usually regulated by some crucial lncRNAs and circRNAs (**Figure 2**).

#### 3.2.4 Other Cancers of Digestive System

In addition to GC, HCC and CRC, miR-149-5p has also been studied in other digestive system cancers, including oral cancer, esophageal cancer and ductal adenocarcinoma of the pancreas (70–76).

According to statistics, more than 140000 people die of oral cancer every year. More than 300000 people are diagnosed with oral cancer each year (77). Studies reported that the expression of miR-149-5p was decreased in oral carcinoma, including tongue squamous cell carcinoma (71) and oral squamous cell carcinoma (OSCC) (75). Luo et al. found that the expression of miR-149-5p in cisplatin-resistant cell line (CAL-27/CDDP) was lower than that in normal OSCC cell line (CAL-27). Functional analysis showed that miR-149-5p could enhance the chemosensitivity of OSCC cells to cisplatin by targeting TGF β2, inhibit cell proliferation, migration and invasion, and promote apoptosis (75). Notably, Qiu et al. confirmed that CircBICD2 knockdown could inhibit the proliferation, migration, invasion, glutamine degradation and increase apoptosis of OSCC cells by regulating the miR-149-5p/IGF2BP1 axis (70). Similarly, down-regulation of lncRNA DLEU1 could inhibit the occurrence of OSCC by regulating the miR-149-5p/CDK6 axis (76).

Esophageal cancer is a global problem and the sixth most common cause of cancer death every year. In 2018, an estimated 572000 people worldwide were diagnosed with esophageal cancer and 509000 died of the disease, indicating a high mortality rate from esophageal cancer (34). The expression of miR-149-5p was low, while LincDRAIC and NFIB were highly expressed in esophageal cancer cells. Down-regulation of DRAIC, NFIB and up-regulation of miR-149-5p can inhibit the proliferation, invasion, and promote apoptosis and autophagy of esophageal cancer cells at the meanwhile. Further studies suggested that DRAIC could act as a sponge for miR-149-5p (72). Consistently, Xu et al. found that miR-149-5p was remarkably down-regulated in esophageal squamous cell carcinoma tissues and cell lines. CIRC_0000654 can be used as a sponge for miR-149-5p to promote the progression of esophageal cancer by indirectly activating IL-6/STAT3 signal pathway (73).

Compared with healthy people and patients with benign pancreatic lesions, the expression of miR-149-5p in serum exosome of patients with pancreatic ductal adenocarcinoma (PDAC) was up-regulated (74). But no further studies elucidated the role of miR-149-5p in PDAC, thus to explore the prognostic and diagnostic role of miR-149-5p, more researches should focus on its function in tumor initiation and progression rather than merely examining its expression level.

### 3.3 Cancers of the Respiratory System

#### 3.3.1 Lung Cancer

Lung cancer remains the most common cancer (11.6% of all cancers) and the leading cause of cancer deaths, with about 1.8 million deaths worldwide in 2018 (78). Histologically, lung cancer is divided into two categories: non-small cell lung cancer (NSCLC) and small cell lung cancer (SCLC), in which NSCLC accounts for more than 80% of lung cancer (79).

**Figure 3:**
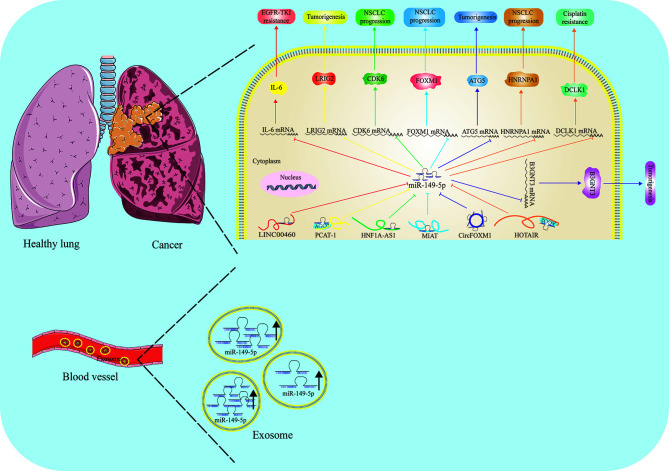
Function and regulatory mechanism of miR-149-5p in lung cancer. The role of miR-149-5p in lung cancer is controversial. In NSCLC, miR-149-5p mostly is regarded as a tumor suppressor by targeting different genes, and is controlled by LINC00460, PCAT-1, HNF1A-AS1, MIAT, HOTAIR and circFOXM1. Paradoxically, high levels of miR-149-5p are found in serum exosome, and promotes LUAD progression.

The role of miR-149-5p in lung cancer is still controversial (**Figure 3**). In 2016, Yang et al. made a comprehensive analysis of the expression profiles of miRNA and mRNA in NSCLC tissues for the first time, and found that the expression of miR-149-5p in cancer tissues was up-regulated compared with that in paracancerous tissues (80). On the contrary, Li et al. found that the expression of miR-149-5p was down-regulated in NSCLC tissues and cell lines. LncRNA PCAT-1 promoted the growth of NSCLC cells by up-regulating LRIG2 through acting as a ceRNA for miR-149-5p, indicating tumor suppressive role of miR-149-5p (81). Consistently, Liu et al. also found that the expression of miR-149-5p was relatively down-regulated in NSCLC. LncRNA HNF1A-AS1, a newly found “oncogene”, was reported to promote the proliferation, migration and invasion of NSCLC cells through sponging with miR-149-5p and targeting CDK6 (82). Zhou et al. found that MIAT can directly bind to miR-149-5p, and then act as a sponge to improve the expression level of FOXM1 and promote the process of NSCLC (83). Li et al. found that HOTAIR can be used as the endogenous ceRNA of miR-149-5p to promote the expression of HNRNPA1, and then promote the proliferation, migration and invasion of NSCLC cells (84). Similarly, Wei et al. found that CircFOXM1 promotes the development of NSCLC by regulating the miR-149-5p/ATG5 axis (85).

Besides, β1, 3-N-acetylglucosaminyltransferase-3(B3GNT3) was reported be abnormally expressed in lung cancer, and the overexpression of B3GNT3 is related to the poor prognosis of patients with lung cancer. Further studies indicated miR-149-5p had a negative regulatory effect on lung cancer progression, and downregulating the expression of B3GNT3 by directly targeting 3’-UTR of B3GNT3. Overexpression of miR-149-5p can antagonize the tumorigenicity of B3GNT3 *in vitro* (86).

Platinum-based chemotherapy after surgical resection has become the standard strategy for the treatment of NSCLC (87). However, the clinical results of patients with NSCLC are still disappointing, mainly due to acquired clinical drug resistance (88). Therefore, reducing drug resistance may be a promising method for the treatment of cisplatin-resistant NSCLC patients.

Zhan et al. found that the expression of lncRNA HOTAIR was up-regulated in DDP-resistant NSCLC tumor tissues and cell lines (A549/DDP and H1299/DDP). The knockdown of HOTAIR decreased the acquired resistance of A549/DDP and H1299/DDP cells to cisplatin, which was characterized by the decrease of 50% inhibitory concentration (IC50) of DDP, the weakening of cell proliferation, migration and invasion *in vitro*, and the inhibition of tumor growth *in vivo*. Functionally, miR-149-5p deletion counteracted the inhibitory effect of HOTAIR gene knockdown on cisplatin resistance; on the contrary, restoring miR-149-5p showed a similar inhibitory effect on cisplatin resistance *in vitro*, and up-regulation of DCLK1 weakened this inhibitory effect. In conclusion, HOTAIR gene knockdown can enhance the cisplatin sensitivity of cisplatin-resistant NSCLC cells partly by regulating the miR-149-5p/DCLK1 axis (89).

Non-small cell lung cancer is a heterogeneous tumor, that can be divided into lung adenocarcinoma(LUAD) and lung squamous cell carcinoma (LUSC) (90). It has been reported that epidermal growth factor receptor tyrosine kinase inhibitor (EGFR-TKIs) is the first choice for the treatment of LUAD with EGFR activation mutation. However, although the initial response to EGFR-TKI treatment was good, most patients eventually developed EGFR-TKI resistance and relapsed within 1 year.

In LUAD cells, LINC00460 promotes EGFR-TKI resistance as a competitive bait for miR-149-5p, thus promoting the expression of IL-6 and inducing EMT-like phenotype. In LUAD cells resistant to gefitinib, the knockdown of LINC00460 restored the response to EGFR-TKI. In addition, compared to patients with low expression of LINC00460 in tumors, patients with high expression of LINC00460 had significantly shorter progression-free survival and shorter overall survival after gefitinib treatment. The discovery of the importance of LINC00460 may lead to its application as a prognostic factor, a diagnostic index of EGFR-TKIs and a molecular target of drugs (91).

Conversely, miR-149-5p was reported be highly expressed in peripheral blood exosome of patients with LUAD. The upregulation of miR-149-5p in exosomes promoted the growth of tumor cells and inhibited the apoptosis of tumor cells. The results of ROC curve analysis showed that exosome miR-149-5p was of good value in the diagnosis of LUAD. Exosome miR-149-5p can directly bind to AMOTL2 and mediate tumor cell proliferation and apoptosis, suggesting that exosome miR-149-5p may be a reliable biomarker of TME in LUAD (17).

Collectively, miR-149-5p may play a tumor suppressive role in NSCLC, and its low expression is usually caused by some oncogenic ceRNAs. However, in LUAD, miR-149-5p is highly expressed in exosome, displaying a tumor promotive role. Therefore, it is important to clarify the role of miR-149-5p in lung cancer, and to detect the exosome miR-149-5p will be more effective to estimate the disease stage.

#### 3.3.2 Nasopharyngeal Carcinoma

Nasopharyngeal carcinoma (NPC) is a type of epithelial carcinoma originating from the lining of nasopharyngeal mucosa. According to the International Agency for Research on Cancer, there were about 129000 new cases of NPC in 2018, accounting for only 0.7 percent of all cancers diagnosed in January 2018. However, its global geographical distribution is extremely uneven, with more than 70% of new cases occurring in East and Southeast Asia (92). At present, radiotherapy, chemotherapy and radiotherapy are the main methods for the treatment of NPC, but some patients always grow to the neck and/or distant metastasis, because of their high metastasis, the prognosis is still very poor (93). Therefore, it is necessary to further study the molecular mechanism of NPC and more effective treatment strategies.

Kong et al. identified a new lncRNA, LINC00460, that is located on chromosome 13q33.2 and is transcribed into a 935nt transcript. The expression of LINC00460 in NPC is significantly higher than that in non-tumor tissues. The overexpression of LINC00460 is closely related to the poor prognosis of patients with NPC. Silencing LINC00460 can inhibit the proliferation of NPC cells *in vivo* and *in vitro*. Mechanistic studies showed that LINC00460 may play an oncogenic role partly by up-regulating the target gene IL-6, acting as a sponge for miR-149-5p in NPC (94). Therefore, the tumor suppressive role of miR-149-5p may be inhibited by LINC00460 in NPC.

### 3.4 Cancers of the Urinary System

#### 3.4.1 Renal Cell Carcinoma

Renal cell carcinoma (RCC) affects more than 400,000 people worldwide every year. The age of diagnosis is about 60 years old, and the number of males is twice as high as that of females (95).

Jin et al. clarified the expression and function of miR-149-5p in RCC for the first time. Compared with normal renal tissue, the expression of miR-149-5p was significantly down-regulated in RCC. Restoring the expression of miR-149-5p with synthetic mimics could inhibit the proliferation and migration and promote apoptosis of RCC cells (96).

The most common subtype of RCC is clear cell renal cell carcinoma(ccRCC) with high morbidity and poor prognosis (97). Okato’s team studied the role of pre-miR-149’s dual strands in ccRCC. The expression level of miR-149-5p in cancer tissues was significantly lower than that in normal tissues, but there was no significant difference in the expression level of miR-149-3p between cancer tissues and non-cancer tissues. The overexpression of miR-149-5p and miR-149-3p could inhibit the proliferation, migration and invasion of renal cancer cells, showing anti-tumor effect. After screening, FOXM1 was the downstream gene of miR-149-5p and miR-149-3p. Compared with normal tissues, the expression of FOXM1 in cancer tissues was significantly up-regulated. In this study, they speculated that miR-149-5p and miR-149-3p jointly regulate the progression and metastasis of ccRCC by acting on FOXM1 (98). These results suggest that miR-149-5p may be a tumor suppressor in RCC.

In addition, Xie et al. constructed a signature containing four-miRNA (miR-21-5p, miR-9-5p, miR-149-5p, and miR-30b-5p), which was related to the survival of ccRCC and can be used as a prognostic biomarker of ccRCC (99). In short, it is crucial for researchers to detect serum miR-149-5p and study whether exosome miR-149-5p is involved in progression of RCC.

#### 3.4.2 Bladder Cancer

Bladder cancer (BC) brings a huge social burden, with more than 500000 new diagnoses and 200000 deaths worldwide every year. The impact of the disease on men (3:1 ratio) is disproportionate to that of the elderly, with a median age of 69 years for men and 71 years for women at the time of diagnosis (100, 101).

CircRNA_100146 was highly expressed in BC, and the increase of circRNA_100146 indicated a poor prognosis of patients with BC. CircRNA_100146 promoted the proliferation, migration and invasion of BC cells through sponging miR-149-5p and promoting the expression of RNF2 (102).

In addition, Lin and other researchers found that the expression level of miR-149-5p in urine of patients with BC were significantly higher than that of healthy controls. The high expression of miR-149-5p was significantly correlated with the overall survival rate of patients with BC, suggesting that urinary miR-149-5p may be a potential biomarker for non-invasive BC detection (103).

### 3.5 Cancers of the Endocrine System

Thyroid cancer (TC) is a common endocrine malignant tumor in the world. It was estimated that there were 567000 confirmed cases and 41000 deaths worldwide in 2018 (34). Thyroid carcinoma is usually divided into differentiated thyroid cancer (DTC), anaplastic thyroid cancer (ATC), medullary thyroid cancer (MTC) (104).

**Figure 4:**
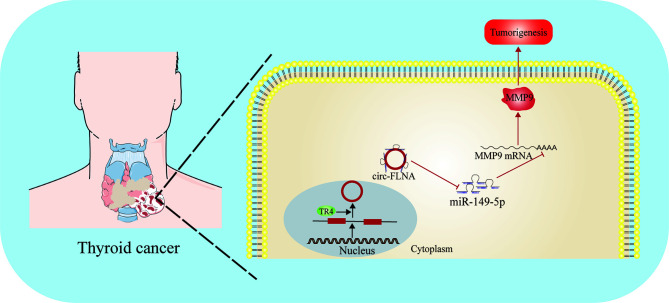
Function and regulatory mechanism of miR-149-5p in thyroid cancer. MiR-149-5p inhibits thyroid cancer progression through targeting MMP9, and is sponged by circFLNA.

Papillary thyroid cancer (PTC) is the most common type of DTC. Although it is generally believed that genetic and environmental factors are related to the occurrence of PTC, the etiology is not completely clear. Rs2292832 is a genetic polymorphism located in the precursor of miR-149. In patients with PTC, compared with TT homozygote and TT/TC combined genotype, the CC genotype of rs2292832 was significantly associated with the increased risk of PTC tumorigenesis and invasion. Interestingly, the expression level of miR-149-5p in PTC patients with CC genotype was lower than that in patients with TC and TT genotypes, suggesting miR-149-5p may function as a tumor suppressor. Rs2292832 may participate in the susceptibility and local progression of PTC in Chinese patients by changing the expression level of miR-149-5p and its target genes (105). A small number of PTC will progress to advanced stage with distant metastasis and have little response to radioiodine therapy or hormone inhibition therapy. Therefore, a better understanding of the molecular biology of metastatic PTC will promote the development of new targeted therapies. Ouyang and his collaborators found that TR4 in PTC patients with distant metastasis was significantly higher than that in patients without metastasis. Mechanism studies have shown that TR4 can directly bind to the upstream promoter of circ-FLNA and regulate its expression at the transcriptional level. circ-FLNA acted as a sponge for miR-149-5p, thereby enhancing the expression of MMP9 and promoting the invasion and migration of PTC cells. In addition, the carcinogenic effect of TR4 and CIRC-FLNA in mouse xenotransplantation model has also been verified. TR4/CIRC-FLNA/miR-149-5p/MMP9 signal may be an ideal therapeutic target for patients with metastatic PTC (106) (**Figure 4**).

Medullary thyroid carcinoma is a malignant tumor originating from parafollicular cells of the thyroid gland, accounting for about 3.8% of TC. The clinical malignant degree of MTC is higher than that of PTC, that is more prone to lymph node metastasis, bringing more difficulties to therapy (107). Ye et al. found that the expression of miR-149-5p in MTC was significantly decreased, and it was significantly correlated with distant metastasis, TNM stage and poor prognosis. Overexpression of miR-149-5p can inhibit the proliferation and invasion of MTC cells. In addition, GIT1 was confirmed to be a directly target for miR-149-5p, and negatively correlated with its expression in MTC. Overexpression of GIT1 could partially reverse the inhibitory effect of miR-149-5p on MTC. Restoring the expression of miR-149-5p by silencing GIT1 may open up a new therapeutic approach for the treatment of MTC (108).

### 3.6 Cancers of the Circulatory System

Recent epidemiological data show that leukemia remains an important cancer worldwide, with about 440000 newly confirmed cases and 310000 deaths in 2018 (34). Leukemia is a blood or bone marrow disease that produces a large number of abnormal white blood cells. There are mainly four types: ALL, AML, CLL and chronic myeloid leukemia (CML) (109).

Among them, AML is the most common malignant tumor, and the overall disease-free recurrence survival rate is about 60%. Tian and his collaborators found that the expression of miR-149-5p was up-regulated in leukemic cell lines and blood and bone marrow samples of leukemia patients, especially in THP-1 cell lines and AML specimens. Fas Ligand (FASLG) was the direct target gene of miR-149-5p and was negatively regulated by miR-149-5p. Mechanism studies have shown that miR-149-5p inhibition induces apoptosis by targeting FASLG, accompanied by the activation of FADD and caspases. In conclusion, inhibition of miR-149-5p may be a potential therapeutic strategy for AML by inducing apoptosis (19).

It has been reported that ALL, a clonal dysplastic disease originating from bone marrow, can produce B-line or T-line lymphocytes, which is common in children. Zhu and his colleagues found that the expression of CircADD2 was down-regulated in bone marrow of children’s ALL and ALL cell lines. Overexpression of CircADD2 could inhibit cell proliferation, and promote cell apoptosis *in vitro* and *in vivo*. Mechanism studies have shown that CircADD2 could directly bind to miR-149-5p and down-regulate the expression level of AKT2, the target gene of miR-149-5p (18).

Collectively, miR-149-5p may play a potential oncogenic role in leukemia by targeting important genes, and is possibly repressed by some important non-coding RNAs. Therefore, it is an effective strategy to reduce the high level of miRNA in leukemia by altering the level of upstream ceRNAs.

### 3.7 Cancers of the Nervous System

Glioma is a primary tumor of the central nervous system, which originates from the inherent constituent cells of the brain, with an estimated annual incidence of 6.6 per 100,000 individuals in the USA (110, 111). Surgery, radiotherapy and temozolomide adjuvant chemotherapy are standard treatments for gliomas; however, due to the resistance to temozolomide, which reduces the cytotoxicity of temozolomide, the prognosis is still poor. Over the past decade, the median survival time of glioma patients has been about 12 months or less (112).

Xu et al. found that the expression of miR-149-5p was significantly down-regulated in glioma cell lines, tumor tissues and leukocytes of glioma patients. Inconsistent with the previous study, glioma patients with miR-149 rs2292832 carrying C allele (CC/CT) have a better prognosis. Functional analysis showed that miR-149 rs2292832C promoted the expression of miR-149-5p, while miR-149-5p could inhibit the proliferation of glioma cells and enhance the killing effect of temozolomide on glioma cells. Further study indicated that CDK6 was the downstream target of miR-149-5p, and miR-149-5p could exert its anticancer effect by inhibiting CDK6/SOX2 pathway. In short, miR-149-5p may be a potential prognostic biomarker of glioma (113).

### 3.8 Cancers of the Motor System

Osteosarcoma is the most common malignant bone tumor. The annual incidence of osteosarcoma was 3.1 ‰, while that of individuals under 25 years old was 4.4 ‰. It is an invasive tumor, which mainly occurs in children and young adults. Although the advances had been made in multidrug chemotherapy and surgical resection of solid tumors, the 5-year overall survival rate and recurrence rate of osteosarcoma patients have not been improved due to tumor metastasis (114).

Xu et al. found that the expression of miR-149-5p was significantly down-regulated in human osteosarcoma and negatively correlated with tumor size, which was an independent prognostic factor for the overall survival of patients with osteosarcoma. The recovery of miR-149-5p expression inhibited the growth of osteosarcoma cells, while knockdown it shows an opposite effect, suggesting that miR-149-5p may be a potential biomarker of prognosis in osteosarcoma patients. Mechanistical studies demonstrated that miR-149-5p inhibits the growth of osteosarcoma cells by regulating the TWEAK/Fn14/PI3K/AKT pathway (115).

## 4 Conclusion

In summary, miR-149-5p displays lower expression level in most cancers, but riches in clinical leukemia samples and peripheral blood of patients with LUAD, which makes a dual role of miR-149-5p in different cancers (**Table 1**). On the one hand, miR-149-5p regulates proliferation, apoptosis, migration, metastasis, and drug-resistance through targeting certain key genes in cancer development. On the other hand, miR-149-5p is regulated by several lncRNAs and circRNAs, such as LINC004600 and CircNRIP1, and its tumor suppressive role is often inhibited by these ceRNAs. These studies revealed a vital role of miR-149-5p in human cancer development, especially reproductive system cancers and digestive system cancers, making it as a promising non-coding RNA for cancer diagnosis, tumor staging and prognosis evaluation.

**Table 1:** MiR-149-5p in different human cancers.

Human System	Tumor type	Upstream regulator	Target gene	Mechanism	Role	References
Reproductive	Breast cancer	NA	MyD88	Reverse PTX resistance	Tumor suppressor	(36)
		NA	IL-6	Reverse Trastuzumab resistance		(38)
		Circ_0072995	SHMT2	Suppress cell malignant properties and anaerobic glycolysis		(39)
		NA	CCT3	Unbalancing the homeostasis in intracellular ROS and the profile of free amino acids in energy metabolism to spur apoptosis		(14)
	Ovarian cancer	NA	MST1 and SAV1	Enhance Cisplatin resistance	Controversy	(41)
		Circ_CELSR1	SIK2	Reverse PTX resistance		(43)
		PVT1	FOXM1	Suppress ovarian cancer cell viability and migration		(44)
	Cervical cancer	Hsa_circ_0075341	AURKA	Weaken the proliferation and invasion of cervical cancer cells	Tumor suppressor	(23)
	Endometrial carcinoma	Hsa_circ_0061140	STAT3	Suppress tumorigenesis	Tumor suppressor	(46)
	Prostate carcinoma	NA	RGS17	Weaken the malignant degree of PCa cells	Tumor suppressor	(48)
		NA	CCT3	Unbalancing the homeostasis in intracellular ROS and the profile of free amino acids in energy metabolism to spur apoptosis		(14)
Digestive	GC	CircNRIP1	AKT1	Inhibit the progression of GC	Tumor suppressor	(13)
		BLACAT1	KIF2A	Inhibit the proliferation, migration and invasion of GC cells		(50)
		CircNHSL1	YWHAZ	Inhibit the progression of GC		(51)
		Circ_0044516	HuR	Inhibit the progression of GC		(52)
	HCC	LINC00461	LRIG2	Inhibit the progression of HCC	Tumor suppressor	(56)
		PART1	MAP2K1	Inhibit proliferation, migration and invasion of HCC cells		(57)
		NEAT1	AKT1	Reverse Sorafenib resistance		(59)
		NA	MMP9	Inhibit the progression of HCC		(15)
	CRC	LINC00460	CUL4A	Inhibit cell proliferation and promote apoptosis	Tumor suppressor	(16)
		LINC00460	BGN	Prevent the metastasis of CRC cells		(22)
		LINC00460	Mutant p53	Reverse Oxaliplatin resistance		(21)
		DLGAP1-AS1	TGFB2	Improve the sensitivity of 5-FU		(67)
		Circ5615	TNKS	Inhibit the proliferation of CRC cells		(68)
		CircCTNNA1	FOXM1	Inhibit CRC progression		(69)
	Oral cancer	NA	TGF β2	Enhance the chemosensitivity of OSCC cells to cisplatin	Tumor suppressor	(75)
		CircBICD2	IGF2BP1	Inhibit OSCC progression		(70)
		DLEU1	CDK6	Inhibit the tumorigenesis of OSCC		(76)
	Esophageal cancer	DRAIC	NFIB	Inhibit the proliferation and invasion as well as promote apoptosis and autophagy of esophageal cancer cells.	Tumor suppressor	(72)
		CIRC_0000654	IL-6	Inhibit the progression of esophageal cancer		(73)
Respiratory	Lung cancer	PCAT-1	LRIG2	Inhibit the development of NSCLC	Controversy	(81)
		HNF1A-AS1	CDK6	Inhibit NSCLC progression		(82)
		MIAT	FOXM1	Inhibit NSCLC progression		(83)
		HOTAIR	HNRNPA1	Inhibit the Cell Growth, Migration and Invasion in NSCLC		(84)
		CircFOXM1	ATG5	Suppress NSCLC development		(85)
		NA	B3GNT3	Antagonize the tumorigenicity		(86)
		HOTAIR	DCLK1	Reverse Cisplatin resistance		(89)
		LINC00460	IL-6	Reverse EGFR-TKI resistance		(91)
		NA	AMOTL2	Promoted the growth and inhibited the apoptosis of tumor cells		(17)
	NPC	LINC00460	IL6	Inhibit the proliferation of NPC cells	Tumor suppressor	(94)
Urinary	RCC	NA	FOXM1	Inhibit the proliferation, migration and invasion of renal cancer cells.	Tumor suppressor	(98)
	BC	CircRNA_100146	RNF2	Inhibit the proliferation, migration and invasion of BC cells	Tumor suppressor	(102)
Endocrine	TC	Circ-FLNA	MMP9	Inhibit the invasion and migration of PTC cells	Tumor suppressor	(106)
		NA	GIT1	Inhibit the proliferation and invasion of MTC cells		(108)
Circulatory	Leukemia	NA	FASLG	Inhibit apoptosis of AML cell	Oncogene	(19)
		CircADD2	AKT2	Promote proliferation and inhibit apoptosis		(18)
Nervous	Glioma	NA	CDK6	Inhibit the proliferation of glioma cells and enhance the killing effect of temozolomide on glioma cells	Tumor suppressor	(113)
Motor	Osteosarcoma	NA	TNFRSF12A	Suppress osteosarcoma cell growth	Tumor suppressor	(115)

NA, not available.

Encouragingly, several studies indicated miR-149-5p displays in exosome, and could be detected in liquid biopsy, such as serum in LUAD and urine in BC, making miR-149-5p be a promising biomarker in evaluation of tumor stage. We hope that future studies should focus more on the collection and analysis of large clinical samples and the levels of miR-149-5p in liquid biopsy. Furthermore, miR-149-5p delivery strategy, such as exosome-based delivery system, could be designed to target tumor tissues in animals first.

Given the role of miR-149-5p in bovine adipogenesis, it is interesting to detect whether miR-149-5p presents in CAAs or CAAs-derived exosomes in different human cancers. Besides, miR-149-5p has been reported to be downregulated in TME of HCC, and increasing studies indicated CAAs are crucial immunomodulators in TME. Therefore, associating miR-149-5p with CAAs in TME is a good research direction, which is more conducive to develop the therapeutic and diagnostic value of miR-149-5p.

## Author Contributions

F-jR, YY, and X-yC drafted the manuscript. Y-tC and QS checked the figures and tables and revised the manuscript. G-yF edited and added the constructive suggestions on the manuscript. All authors contributed to the article and approved the submitted version.

## Conflict of Interest

The authors declare that the research was conducted in the absence of any commercial or financial relationships that could be construed as a potential conflict of interest.

## Publisher’s Note

All claims expressed in this article are solely those of the authors and do not necessarily represent those of their affiliated organizations, or those of the publisher, the editors and the reviewers. Any product that may be evaluated in this article, or claim that may be made by its manufacturer, is not guaranteed or endorsed by the publisher.
